# Retraction: Palladium complex supported on the surface of magnetic Fe_3_O_4_ nanoparticles: an ecofriendly catalyst for carbonylative Suzuki-coupling reactions

**DOI:** 10.1039/d5ra90061a

**Published:** 2025-05-15

**Authors:** Shanshan Jiang

**Affiliations:** a Department of Chemistry and Chemical Engineering, Lvliang University Lvliang Shanxi 033000 PR China jiang_shanshan1@sina.com

## Abstract

Retraction of ‘Palladium complex supported on the surface of magnetic Fe_3_O_4_ nanoparticles: an ecofriendly catalyst for carbonylative Suzuki-coupling reactions’ by Shanshan Jiang, *RSC Adv.*, 2023, **13**, 34273–34290, https://doi.org/10.1039/D3RA06533B.

The Royal Society of Chemistry hereby wholly retracts this *RSC Advances* article due to concerns with the reliability of the data.

Multiple NMR spectra show signs of manipulation and duplication with other papers by different authors.^1,2^ The author was contacted but did not provide a response to the concerns.

Given the significance of these concerns, the Editor has lost confidence that the findings presented in this paper are reliable.

The author was informed about the retraction of the article but has not responded.

Signed: Laura Fisher, Executive Editor, *RSC Advances*

Date: 9th May 2025
